# Supplementary material for: Double-phase metasurface operators for all-optical image processing
Source: Light Sci Appl. 2026 Feb 23;15:119. doi: 10.1038/s41377-025-02153-w (PMC12929636; doi:10.1038/s41377-025-02153-w)
Supplement: Supplementary file 1 — Supplementary Information [file 41377_2025_2153_MOESM1_ESM.pdf]

Supplementary Information for Double-phase metasurface operators for all-optical image processing

Linzhi Yu[1], Haobijam J. Singh[1], Jesse Pietila[1], Humeyra Caglayan[1,2*]

[1*]Department of Physics, Tampere University, Tampere, 33720, Finland.
[2]Department of Electrical Engineering and Eindhoven Hendrik Casimir Institute, Eindhoven University of Technology, Eindhoven, 5600 MB, The Netherlands.

*Corresponding author(s). E-mail(s): h.caglayan@tue.nl;
Contributing authors: linzhi.yu@tuni.fi; johnson.singh@tuni.fi; jesse.pietila@tuni.fi;

Supplementary Contents

1. Design workflow of meta-operators and complex meta-holograms 3

2. Supplementary simulations and experimental validation of meta-Kernels for all-optical image processing 4

3. Design and simulation methods of complex meta-holograms 8

4. Supplementary simulations and experimental verification of complex meta-holograms 10

5. Meta-atom library design and metasurface error analysis 12

6. Spatially resolved transmission measurements of metasurfaces based on linear polarization multiplexing 19

7. Design of PB phase meta-atoms 21

8. Meta-operators' robustness to polarization misalignment 23

9. Broadband performance of meta-operators based on circular polarization multiplexing 25

10. Experiment setups 27

11. TiO_2 metasurfaces fabrication 29

12. Test samples fabrication 30

13. Metalens-based $4f$ configuration for planar optical processor 32

14. Comparison with representative metasurface-based approaches for analog image processing 33

Video 1. Volumetric spiral dot array hologram

Video 2. Volumetric *TAU* letters hologram

1. Design workflow of meta-operators and complex meta-holograms

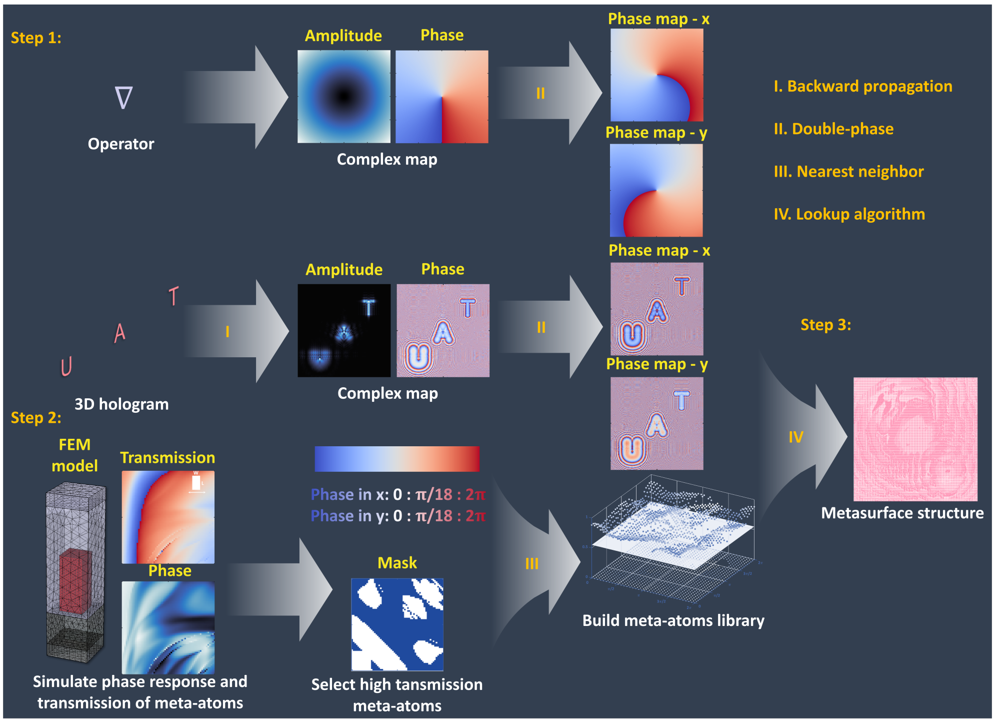

Fig. S1 Workflow for designing meta-operators and complex meta-holograms.

The design workflow for meta-operators and meta-holograms, illustrated in Fig. S1, consists of three main steps. First, for meta-operators, the desired complex transfer functions in the Fourier domain are analytically designed and converted into phase distributions using double-phase encoding. For meta-holograms, the target volumetric intensity distribution is back-propagated to the meta-hologram plane to obtain the corresponding complex interference field, which is then encoded using the same double-phase encoding method (details in Section 3). Next, a meta-atom library is generated by simulating the transmission spectra of meta-atoms with varying geometries using the finite-difference frequency-domain (FDFD) method. Meta-atoms with high transmission efficiency are selected, and the target phase shifts, spanning $[0, 2\pi)$, are discretized into 36 levels per polarization state, resulting in 1296 unique configurations. A nearest-neighbor algorithm is then applied to match each required phase shift to the most suitable meta-atom structure (details in Algorithm S1, Section 5). Finally, the designed meta-operators and complex meta-holograms are physically realized by mapping the computed phase distributions onto the corresponding meta-atoms using a lookup algorithm (details in Algorithm S2, Section 5).

2. Supplementary simulations and experimental validation of meta-Kernels for all-optical image processing

Figs. S2 and S3 show the amplitude and phase distributions of the complex transfer functions for meta-operators in the Fourier domain, along with the corresponding theoretical intensity distributions of the processed images for comparison with experimental results. These theoretical intensity distributions are simulated using Fourier optics [1]. The simulation process begins by computing the amplitude transfer functions (ATFs) of the meta-operators for different polarization states. The ATFs are then applied to the spatial spectrum of the input light field to generate the corresponding output spatial spectrum. The final intensity distributions are obtained by taking the absolute square of the interfered output fields. Fig. S4 presents additional experimental results showcasing the performance of various meta-operators with different input images.

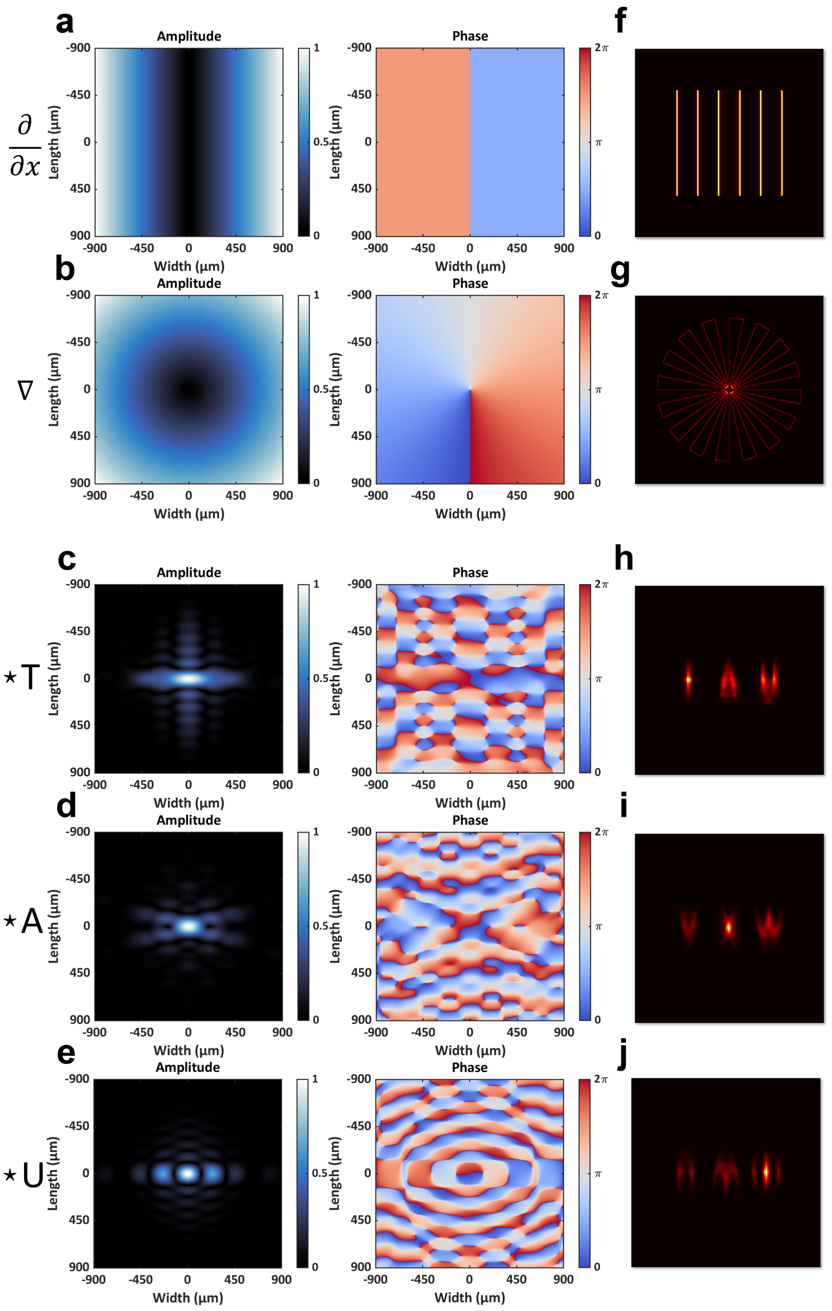

Fig. S2 Amplitude and phase distributions of the complex transfer functions for meta-operators implemented via linear polarization multiplexing, along with the corresponding theoretical intensity distributions of the processed images for comparison with experimental results in the Main Text.

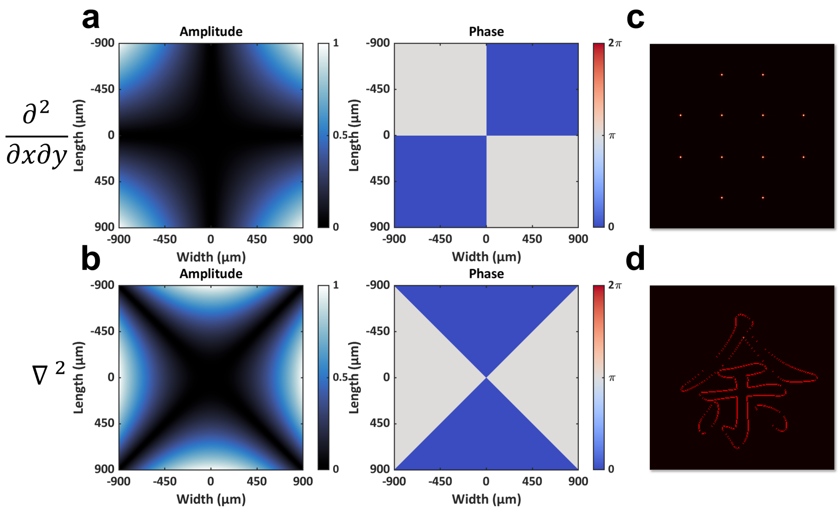

Fig. S3 **Amplitude and phase distributions of the complex transfer functions for meta-operators implemented via circular polarization multiplexing, along with the corresponding theoretical intensity distributions of the processed images for comparison with experimental results in the Main Text.**

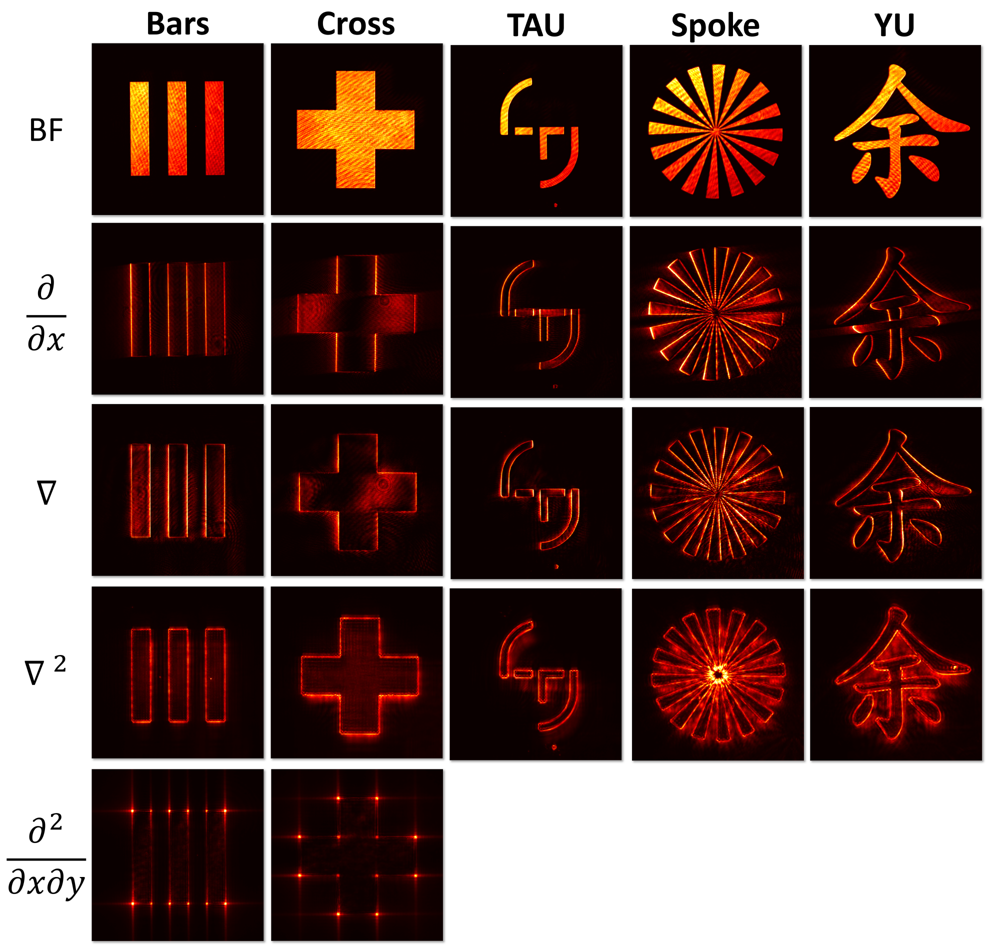

Fig. S4 **Additional experimental results showing images captured under bright-field (BF) illumination and their corresponding outputs after processing with meta-operators.**

3. Design and simulation methods of complex meta-holograms

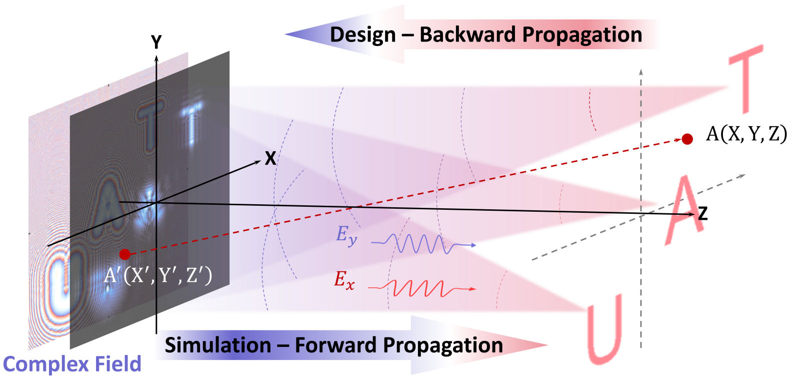

Fig. S5 Schematic illustration of the back-propagation method used for designing complex meta-holograms and the forward propagation process for simulating the light field intensity distribution in space after modulation by the meta-holograms.

The design and simulation process for the complex meta-hologram is illustrated in Fig. S5. In the design stage, each point of the target volumetric hologram is treated as a point source with an initial phase of zero, represented as $E(x, y, z)$. At this stage, the polarization of the electromagnetic field is not considered. By back-propagating light from each point to the meta-hologram plane, a coherent interference field is obtained, defining the complex amplitude modulation required for the meta-hologram. This complex amplitude is then encoded into phase distributions for x- and y-polarizations using the double-phase encoding method. The propagation of the light field in space is modeled using the angular spectrum method [1, 2], which decomposes the optical field into a superposition of plane waves. The process begins by applying a Fourier transform to compute the angular spectrum of the field:

$$A\left(\frac{\alpha}{\lambda}, \frac{\beta}{\lambda}; z\right) = \iint E(x, y, z) \exp\left[-j\frac{2\pi}{\lambda}(\alpha x + \beta y)\right] dxdy \tag{S1}$$

To simulate backward propagation to the meta-hologram plane ($z = 0$), the transfer function is applied:

$$H_{\text{backward}} = \exp\left[-j\frac{2\pi}{\lambda} z\sqrt{1 - \alpha^2 - \beta^2}\right] \tag{S2}$$

$$A'\left(\frac{\alpha}{\lambda}, \frac{\beta}{\lambda}; 0\right) = A\left(\frac{\alpha}{\lambda}, \frac{\beta}{\lambda}; z\right) \cdot H_{\text{backward}} \tag{S3}$$

The field at the meta-hologram plane is then retrieved using an inverse Fourier transform:

$$E'(x', y', 0) = \iint A'\left(\frac{\alpha}{\lambda}, \frac{\beta}{\lambda}; 0\right) \exp\left[j\frac{2\pi}{\lambda}(\alpha x' + \beta y')\right] d\frac{\alpha}{\lambda} d\frac{\beta}{\lambda} \tag{S4}$$

To simulate forward propagation, the backward propagation transfer function H_{backward} is replaced with its inverse H_{forward}, enabling the computation of light fields at arbitrary depths. For birefringent meta-holograms, the x- and y-polarization components are processed separately. The complex field at $z = 0$ is given by $\begin{bmatrix} E'_x \\ E'_y \end{bmatrix}$, with corresponding angular spectra:

$$\left[A'_x \ A'_y\right] = \iint_{-\infty}^{\infty} \left[E'_x \ E'_y\right] \exp\left[-j\frac{2\pi}{\lambda}\left(\alpha x' + \beta y'\right)\right] dx'dy' \tag{S5}$$

Applying the forward transfer function:

$$H_{\text{forward}} = H_{\text{backward}}^{-1} \tag{S6}$$

$$\begin{bmatrix} A_x \\ A_y \end{bmatrix} = \begin{bmatrix} A'_x \\ A'_y \end{bmatrix} \cdot H_{\text{forward}} \tag{S7}$$

The field at a given depth z is then obtained via inverse Fourier transform:

$$\left[E_x \ E_y\right] = \iint_{-\infty}^{\infty} \left[A_x \ A_y\right] \exp\left[j\frac{2\pi}{\lambda}\left(\alpha x + \beta y\right)\right] d\frac{\alpha}{\lambda} d\frac{\beta}{\lambda} \tag{S8}$$

The reconstructed intensity is determined by the coherent interference of x- and y-polarization components:

$$I(x, y, z) = \left(\frac{\sqrt{2}}{2}E_x + \frac{\sqrt{2}}{2}E_y\right) \cdot \text{conj}\left(\frac{\sqrt{2}}{2}E_x + \frac{\sqrt{2}}{2}E_y\right) \tag{S9}$$

When determining the optimal transmission threshold for constructing the meta-atom library, a 2D hologram is simulated while accounting for phase shift approximation errors and the non-uniform transmission of meta-atoms. The near-field amplitude and phase shifts for both polarization states are extracted from finite element simulations. The final hologram is then reconstructed using the angular spectrum method.

4. Supplementary simulations and experimental verification of complex meta-holograms

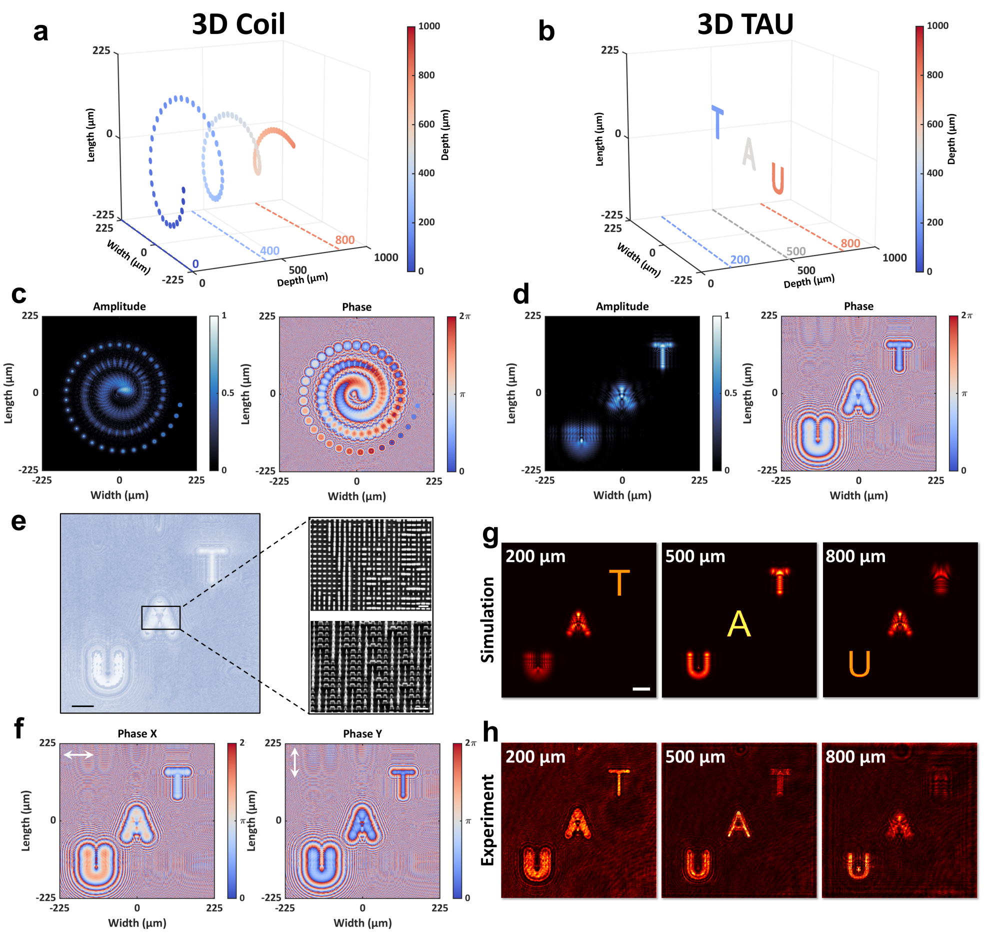

Fig. S6 Supplementary experimental results of complex meta-holograms. (a) Designed volumetric spiral dot array hologram, with dots uniformly distributed from the meta-hologram plane to a depth of 800 μm. (b) Designed volumetric *TAU* letters hologram, where the letters *T*, *A*, and *U* are positioned at depths of 200 μm, 500 μm, and 800 μm, respectively. (c) Amplitude and phase distributions of the complex volumetric spiral dot array hologram. (d) Amplitude and phase distributions of the complex *TAU* hologram. (e) Optical and SEM images of the fabricated volumetric *TAU* meta-hologram. (f) Phase maps of the volumetric *TAU* meta-hologram under x- and y-polarized light. (g, h) Simulated and experimentally measured light field intensities of the volumetric *TAU* hologram at different depths in space. Scale bars: optical images, 50 μm; SEM images, 1 μm; hologram images, 50 μm.

A complex meta-hologram displaying the word TAU was designed, with the letters T, A, and U positioned at depths of 200 µm, 500 µm, and 800 µm from the meta-hologram plane, respectively, as shown in Fig. S6b. The meta-hologram consists of 1000 $\times$ 1000 meta-atoms, corresponding to a physical size of 450 µm $\times$ 450 µm. The hologram was initially generated as a complex-amplitude distribution using the backward propagation method, as shown in Fig. S6d. These distributions were then encoded into phase maps for x- and y-polarized light via double-phase encoding, depicted in Fig. S6f. Optical and SEM images of the fabricated meta-hologram are presented in Fig. S6e. The simulated intensity distributions at different depths in space are shown in Fig. S6g, with the corresponding experimental results in Fig. S6h, demonstrating strong agreement between simulation and experiment.

5. Meta-atom library design and metasurface error analysis

To construct the meta-atom library, we begin by simulating the phase shift and transmission efficiency of rectangular nanopillars under x- and y-polarized light using the FDFD method in the COMSOL Multiphysics RF Module. Each nanopillar is characterized by its length and width, which determine its optical properties. Nanopillars with high transmission efficiency are selected as candidate units based on a transmission threshold, ensuring a balance between transmission uniformity and phase coverage. The optimal threshold is determined by analyzing the trade-off between transmission efficiency and phase fidelity, considering material and structural parameters.

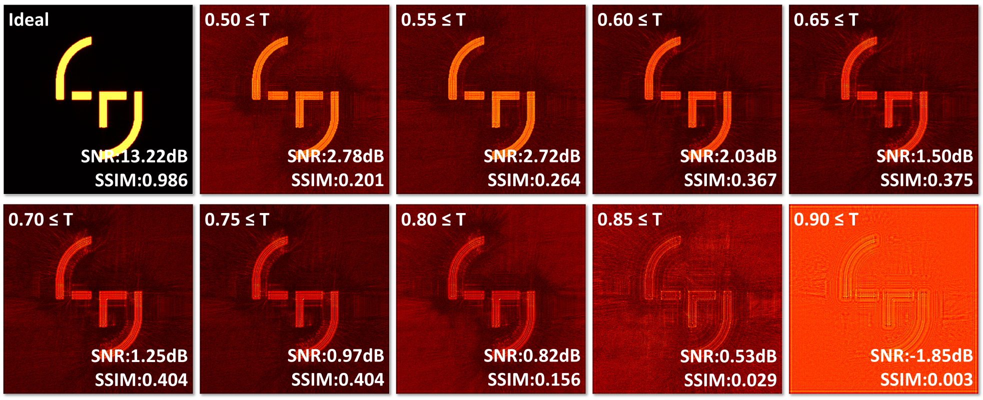

Fig. S7 Comparison of holograms generated using meta-holograms constructed from meta-atom libraries with transmission thresholds (T) ranging from 0.50 to 0.90. The ideal hologram (top-left) represents a case with uniform transmission and no phase errors. For each threshold, the corresponding SNR and SSIM are provided. The optimal threshold is determined as $T = 0.55$, achieving a balance between transmission efficiency and hologram quality.

To evaluate the impact of the transmission threshold on hologram performance, Fig. S7 presents a comparative analysis of a 2D hologram displaying the Tampere University logo (Fig. S25d), generated using meta-holograms constructed from meta-atom libraries with transmission thresholds ranging from 0.50 to 0.90. The ideal hologram (top-left) represents a case with uniform transmission and no phase errors. For each threshold, the corresponding signal-to-noise ratio (SNR) and structural similarity index (SSIM) are provided [3]. Based on both quantitative criteria and visual assessment, we set the threshold at $T = 0.55$, achieving an optimal balance between transmission efficiency and hologram quality. Notably, materials with improved optical properties could further enhance transmission uniformity and reduce phase shift errors, offering opportunities for higher-performance meta-operators and meta-holograms.

Algorithm S1 Meta-Atom Library Generation via Nearest-Neighbor Matching

Input:

$D = \{\phi_x^{\text{real}}, \phi_y^{\text{real}}, T_x, T_y, l_x, l_y\}$: Dataset with dimensions (l_x, l_y), transmission T_x, T_y, and phase shift $\phi_x^{\text{real}}, \phi_y^{\text{real}}$.

$T_{\text{threshold}}$: Transmission threshold.

n: Number of target phase samples.

Output:

$L = \{\phi_x^{\text{target}}, \phi_y^{\text{target}}, \phi_x^{\text{real}}, \phi_y^{\text{real}}, T_x, T_y, l_x, l_y\}$: Library dataset with target and real phases, transmission, and dimensions.

Pseudocode:

$D_{\text{high}} \leftarrow D(T_x, T_y > T_{\text{threshold}})$

$L \leftarrow \emptyset$

for $i \in \{1, \dots, n\}$ **do**

  for $j \in \{1, \dots, n\}$ **do**

    $(\phi_{x,i}^{\text{target}}, \phi_{y,j}^{\text{target}}) \leftarrow \left(\frac{i \cdot 2\pi}{n}, \frac{j \cdot 2\pi}{n}\right)$

    for all D_{high} **do**

      $\text{distances} = \sqrt{(D_{\text{high}}(\phi_x^{\text{real}}) - \phi_{x,i}^{\text{target}})^2 + (D_{\text{high}}(\phi_y^{\text{real}}) - \phi_{y,j}^{\text{target}})^2}$

    end for

    $L \leftarrow L \cup \{\phi_{x,i}^{\text{target}}, \phi_{y,j}^{\text{target}}, D_{\text{high,min(distances)}}(\phi_x^{\text{real}}, \phi_y^{\text{real}}, T_x, T_y, l_x, l_y)\}$

  end for

end for

return L

Applying this threshold, we construct the meta-atom library by selecting nanopillars with transmission above $T = 0.55$, as illustrated by the transmission mask in Fig. S8a. In this study, we adopt $n = 36$ discrete phase levels for both x- and y-polarizations, covering a phase range from 0 to 2π with a resolution of $\pi/18$ per phase level. Each discrete target phase pair $(\phi_x^{\text{target}}, \phi_y^{\text{target}})$, is mapped to a nanopillar with corresponding length and width using a nearest-neighbor algorithm. The nearest-neighbor algorithm identifies the nanopillar whose phase shifts, $(\phi_x^{\text{real}}, \phi_y^{\text{real}})$, minimize the error relative to the target phase. Specifically, the nanopillar with the smallest phase shift deviation is selected, where the error is quantified by a distance metric (loss function) defined as:

$$\text{distance} = \sqrt{\left(\phi_x^{\text{real}} - \phi_x^{\text{target}}\right)^2 + \left(\phi_y^{\text{real}} - \phi_y^{\text{target}}\right)^2} \tag{S10}$$

The pseudocode for the nearest-neighbor algorithm is provided in Algorithm S1. The resulting meta-atom library comprises 1296 distinct meta-atoms, visualized in Fig. S8b.

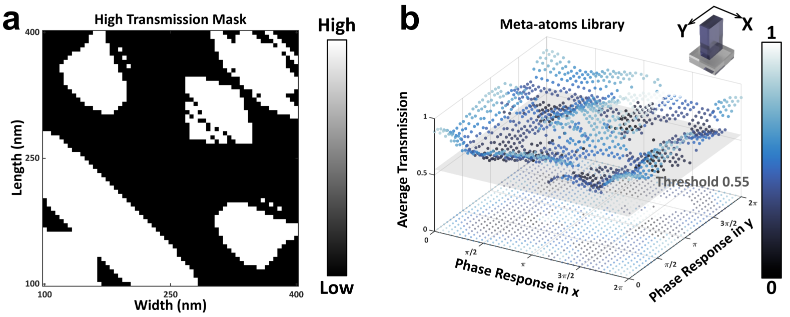

Fig. S8 Design of the meta-atom library. (a) Transmission mask applied to select high-efficiency meta-atoms using a threshold of $T = 0.55$. (b) Three-dimensional visualization of the meta-atom library, depicting the phase shift distribution and corresponding average transmission for x- and y-polarized illumination.

Once the meta-atom library is established, a lookup algorithm (Algorithm S2) maps the target phase distributions to the meta-atoms that minimize phase error, enabling the metasurfaces to closely reproduce the desired optical response while maintaining high transmission efficiency. The simulated transmission and phase characteristics for meta-operators based on linear polarization multiplexing and for complex meta-holograms are shown in Fig. S9 and Fig. S10, respectively. The corresponding experimental transmission measurements are provided in Section 6, confirming agreement between the simulations and the fabricated devices.

To assess the impact of deviations in the selected meta-atom responses, including phase error from discretization and nonuniform amplitude transmission, additional simulations were performed using the library's complex transmission. The resulting output intensity distributions for the meta-operators and the meta-holograms are presented in Fig. S11. Compared with the ideal simulations in Fig. S2, Fig. 6 (Main Text), and Fig. S6, where the phase error and nonuniform transmission are not considered, the performance remains robust to these design nonidealities. For the meta-operators, salient image features are retained with only slight reductions in contrast. For the meta-holograms, the reconstructions show moderate contrast loss but preserve the intended intensity distributions across depths. For the meta-holograms, the effect of these nonidealities on the reconstructed field is further illustrated in Fig. S12, which shows simulated intensity cross sections along the optical axis.

Algorithm S2 Efficient Meta-Atom Mapping via Lookup Algorithm for Metasurface Fabrication

Input:

$\phi_x^{\text{target}}, \phi_y^{\text{target}}$: Target phase in the x- and y-directions with size (m, n).
$L = \{\phi_x^{\text{lib}}, \phi_y^{\text{lib}}, T_x^{\text{lib}}, T_y^{\text{lib}}, l_x^{\text{lib}}, l_y^{\text{lib}}\}$: Library of meta-atoms with phases, transmissions, and dimensions.

Output:

$M = \{\phi_x^{\text{mapped}}, \phi_y^{\text{mapped}}, T_x^{\text{mapped}}, T_y^{\text{mapped}}, l_x^{\text{mapped}}, l_y^{\text{mapped}}\}$: Mapped library of meta-atoms with phases, transmissions, and dimensions.

Pseudocode:

Initialize $M = \emptyset$
for $i \in \{1, \ldots, m\}$ **do**
    for $j \in \{1, \ldots, n\}$ **do**
        for all L **do**
            errors $= \sqrt{(L(\phi_x^{\text{lib}}) - \phi_{x,(i,j)}^{\text{target}})^2 + (L(\phi_y^{\text{lib}}) - \phi_{y,(i,j)}^{\text{target}})^2}$
        end for
        $M_{(i,j)} \leftarrow L_{\min(\text{errors})}\{\phi_x^{\text{lib}}, \phi_y^{\text{lib}}, T_x^{\text{lib}}, T_y^{\text{lib}}, l_x^{\text{lib}}, l_y^{\text{lib}}\}$
    end for
end for

return M

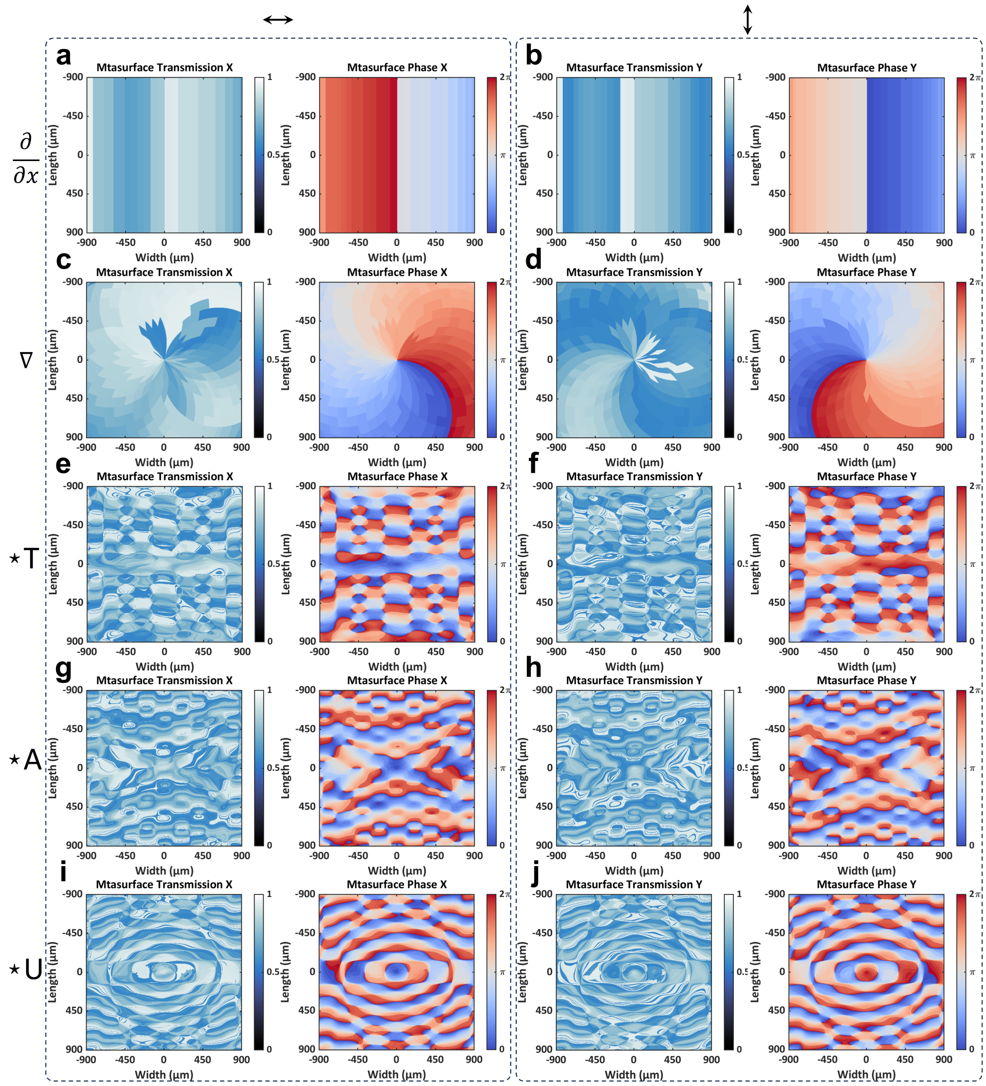

Fig. S9 Transmission and phase shift characteristics for x- and y-polarized incident light of meta-operators based on linear polarization multiplexing, designed using the meta-atom library in Fig. S8.

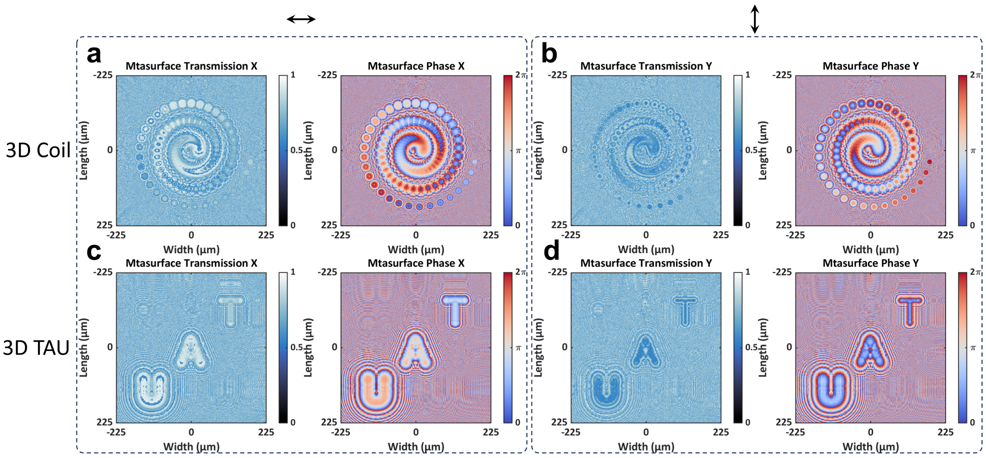

Fig. S10 **Transmission and phase shift characteristics of complex meta-holograms for x- and y-polarized incident light, designed using the meta-atom library in Fig. S8.**

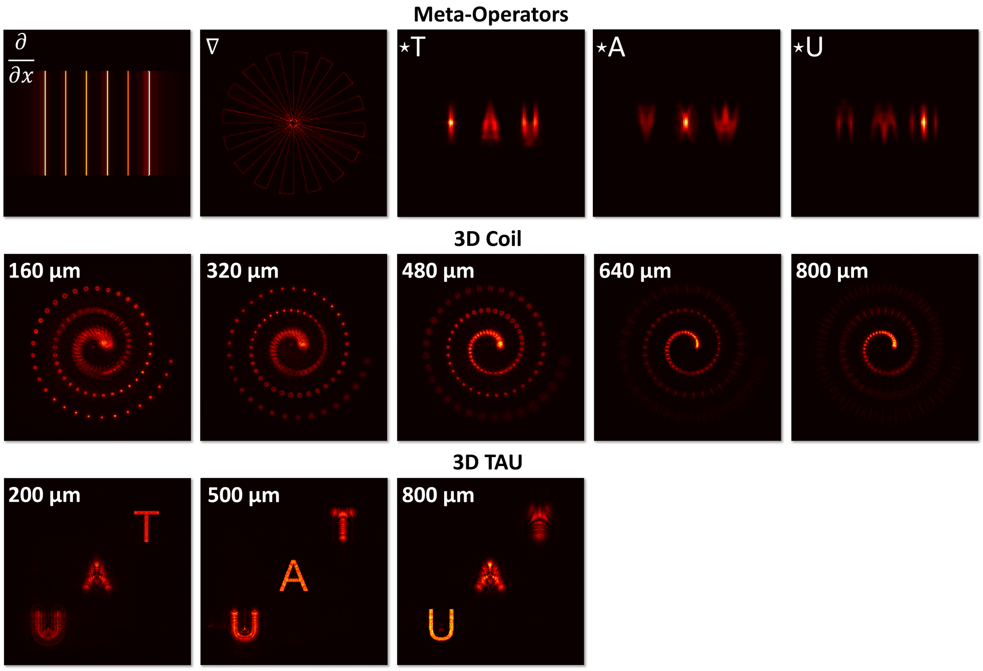

Fig. S11 **Numerical evaluation of metasurface performance with complex transmission response.**

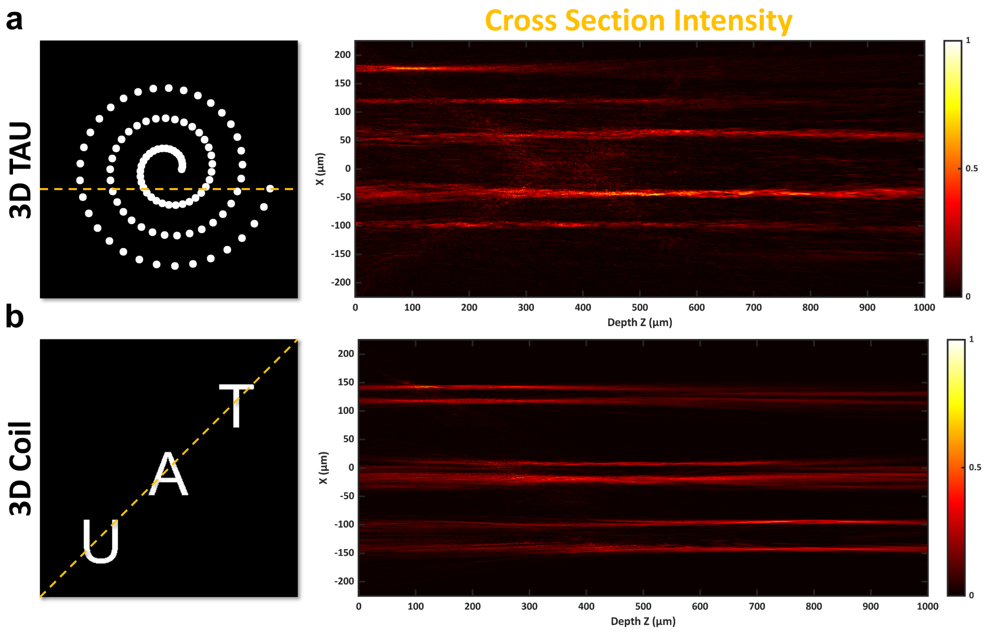

Fig. S12 Simulated cross-sectional intensity of reconstructed holograms. (a) Volumetric spiral-dot array hologram. (b) Volumetric *TAU* letters hologram. Simulated optical-field intensity cross sections defined by the optical axis and the dashed line.

6. Spatially resolved transmission measurements of metasurfaces based on linear polarization multiplexing

To provide an additional experimental reference for consistency between design and fabrication, we measured the spatially resolved transmission of metasurfaces based on linear polarization multiplexing, in which the local transmission varies with the meta-atom configuration.

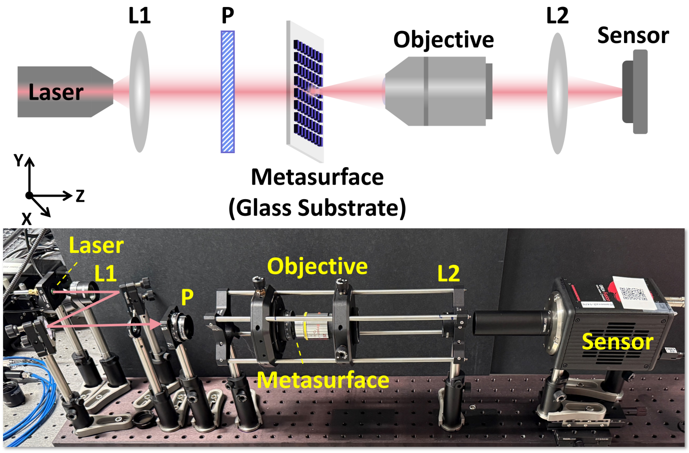

Fig. S13 Optical setup for transmission measurements. Metasurfaces based on linear polarization multiplexing measured under x- and y-polarized illumination.

The transmission measurement setup is shown in Fig. S13. A 532 nm laser was expanded and collimated with an achromatic lens (L1, AC254-030-A, Thorlabs) and then passed through a linear polarizer (P1, LPVISE100-A, Thorlabs) to generate x- or y- polarized illumination. The sample (metasurface or bare-glass reference) was imaged onto a camera (ORCA-Fusion C14440-20UP, Hamamatsu) using an objective (EC EPIPLAN-NEOFLUAR 2.5×/0.06, ZEISS for meta-operators; EC EPIPLAN-NEOFLUAR 10×/0.25, ZEISS for meta-holograms) and a tube lens (L2, AC254-150-A, Thorlabs). Per-pixel transmission T was computed as

$$T = \frac{I_{\text{meta}} - I_{\text{dark}}}{I_{\text{ref}} - I_{\text{dark}}} \tag{S11}$$

where I_{meta} is the recorded intensity from the metasurface, I_{ref} is the intensity from the bare-glass reference under identical settings, and I_{dark} is the background signal recorded without illumination.

The measured transmission maps under x- and y-polarized illumination are presented in Fig. S14 for the meta-operators and Fig. S15 for the meta-holograms. The measurements agree well with the simulated design expectations and reproduce the intended spatial variation. Some deviations are attributable to fabrication tolerances, such as small variations in nanopillar dimensions, and residual conductive polymer from scanning electron microscopy imaging that can locally affect transmission. Speckle and source-intensity fluctuations inherent to coherent illumination also

contribute to background nonuniformity. Despite these factors, the measured transmission maps provide a useful reference for assessing metasurface quality and confirm consistency with the design.

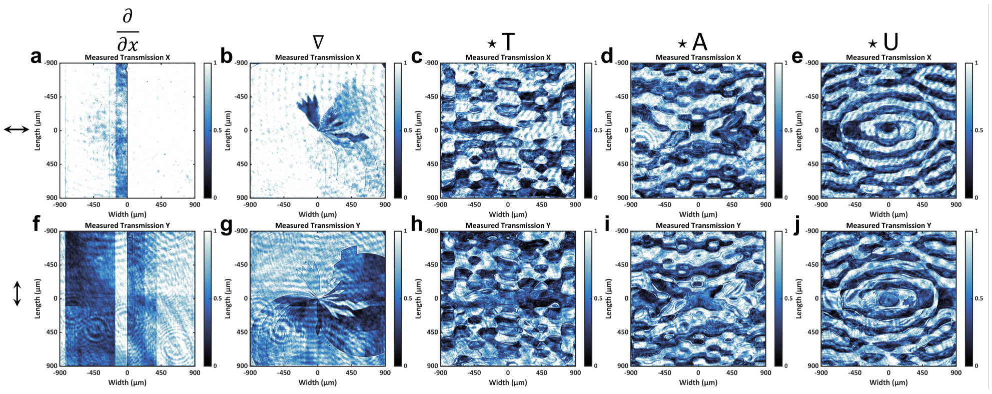

Fig. S14 Transmission maps of meta-operators. Measured under x- and y-polarized illumination at 532 nm.

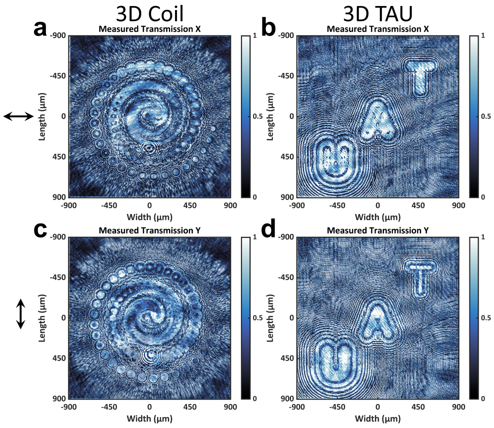

Fig. S15 Transmission maps of meta-holograms. Measured under x- and y-polarized illumination at 532 nm.

7. Design of PB phase meta-atoms

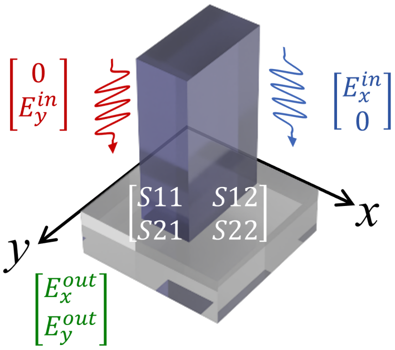

Fig. S16 Schematic illustration of a rectangular TiO_2 nanopillar meta-atom under incident electric fields, demonstrating its interaction with x- and y-polarized light.

The meta-operators based on circular polarization multiplexing in this study consist of Pancharatnam-Berry (PB) phase meta-atoms, which function as half-waveplates to achieve high-efficiency cross-polarization conversion at the operational wavelength of 532 nm. These meta-atoms are realized using rectangular TiO_2 nanopillars with a fixed height of 600 nm, while their length and width are optimized to maximize conversion efficiency. To determine the optimal nanopillar dimensions, we perform FDFD simulations to extract the complex transmission coefficients, represented by the $S21$ scattering parameters for x- and y-polarized light, denoted as $S21_x$ and $S21_y$, respectively. These parameters are then transformed into their corresponding values for right circularly polarized (RCP) and left circularly polarized (LCP) light using:

$$\begin{aligned} S21_L &= \frac{S21_x - iS21_y}{\sqrt{2}} \\ S21_R &= \frac{S21_x + iS21_y}{\sqrt{2}} \end{aligned} \tag{S12}$$

The S_{21} parameters for cross-polarization conversion, denoted as S_{21L2R} (LCP to RCP) and S_{21R2L} (RCP to LCP), are derived as follows:

$$\begin{aligned} S_{21L2R} &= \frac{iS_{21L_y} - iS_{21L_x}}{\sqrt{2}} \\ S_{21R2L} &= \frac{iS_{21R_y} + iS_{21R_x}}{\sqrt{2}} \end{aligned} \tag{S13}$$

where S_{21L_x}, S_{21L_y} are the x- and y-components of S_{21L}, and S_{21R_x}, S_{21R_y} are the x- and y-components of S_{21R}. The cross-polarization conversion efficiencies for LCP-to-RCP (η_{L2R}) and RCP-to-LCP (η_{R2L}) transformations are then calculated as:

$$\eta_{L2R} = |S21_{L2R}|^2$$
$$\eta_{R2L} = |S21_{R2L}|^2 \tag{S14}$$

Based on these calculations, the optimal nanopillar dimensions were determined to be 235 nm in length and 115 nm in width. Each nanopillar is oriented along its short axis according to the designed phase distribution to achieve the desired phase control, ensuring efficient phase modulation.

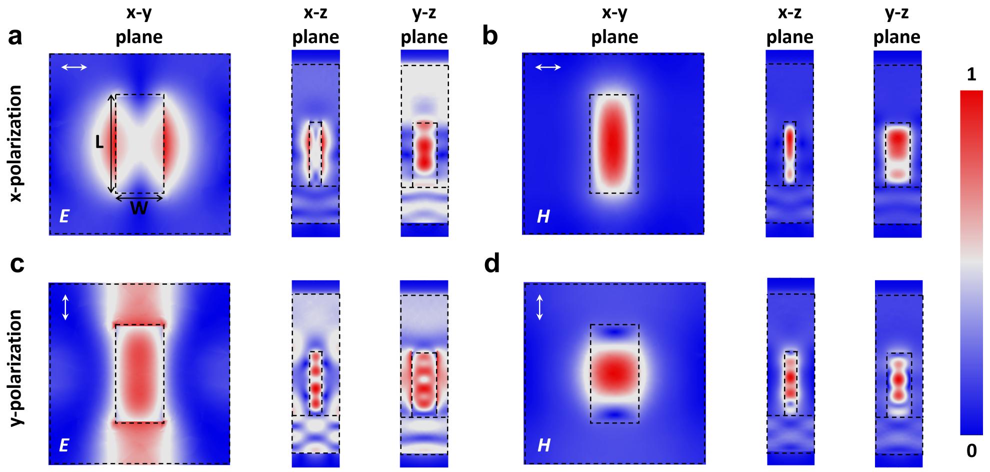

Fig. S17 Simulated energy density distribution of TiO_2 nanopillars under a linearly polarized plane wave at 532 nm.

Fig. S17 presents the simulated electric and magnetic field energy distributions of the optimized TiO_2 nanopillars. The strong field confinement within the nanopillars confirms their role as independent half-wave plates, enabling precise local PB phase modulation for meta-operators.

8. Meta-operators' robustness to polarization misalignment

The performance of meta-operators can be influenced by misalignment between the input and output polarizers. To evaluate robustness under such deviations, a simulation analysis was conducted to quantify the effect of analyzer angle error on output quality.

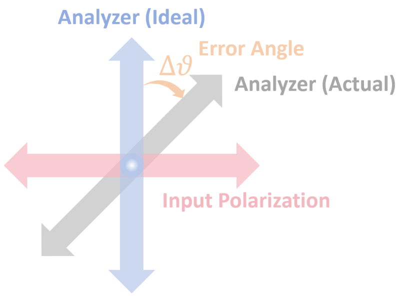

Fig. S18 Schematic of polarization misalignment. Definition of the analyzer-angle deviation $\Delta\vartheta$ with respect to the ideal setting.

As shown in Fig. S18, the analyzer angle error $\Delta\vartheta$ was varied from 0° to 10° with the input polarizer fixed. Representative meta-operators were applied to the same test images used in the Main Text, and the resulting outputs are presented in Fig. S19. As $\Delta\vartheta$ increases, the output images exhibit a progressive reduction in contrast, an increase in background intensity, and reduced edge acutance. Despite this degradation, key features of the processed patterns remain clearly distinguishable even at $\Delta\vartheta = 10°$. Among the tested operators, the cross-correlation cases targeting the letters T, A, and U maintain reliable pattern recognition across the range, whereas the Laplacian operator is comparatively more sensitive to misalignment.

These results indicate that, although sensitivity varies across operators, the metasurface platform maintains reliable performance within typical experimental alignment tolerances.

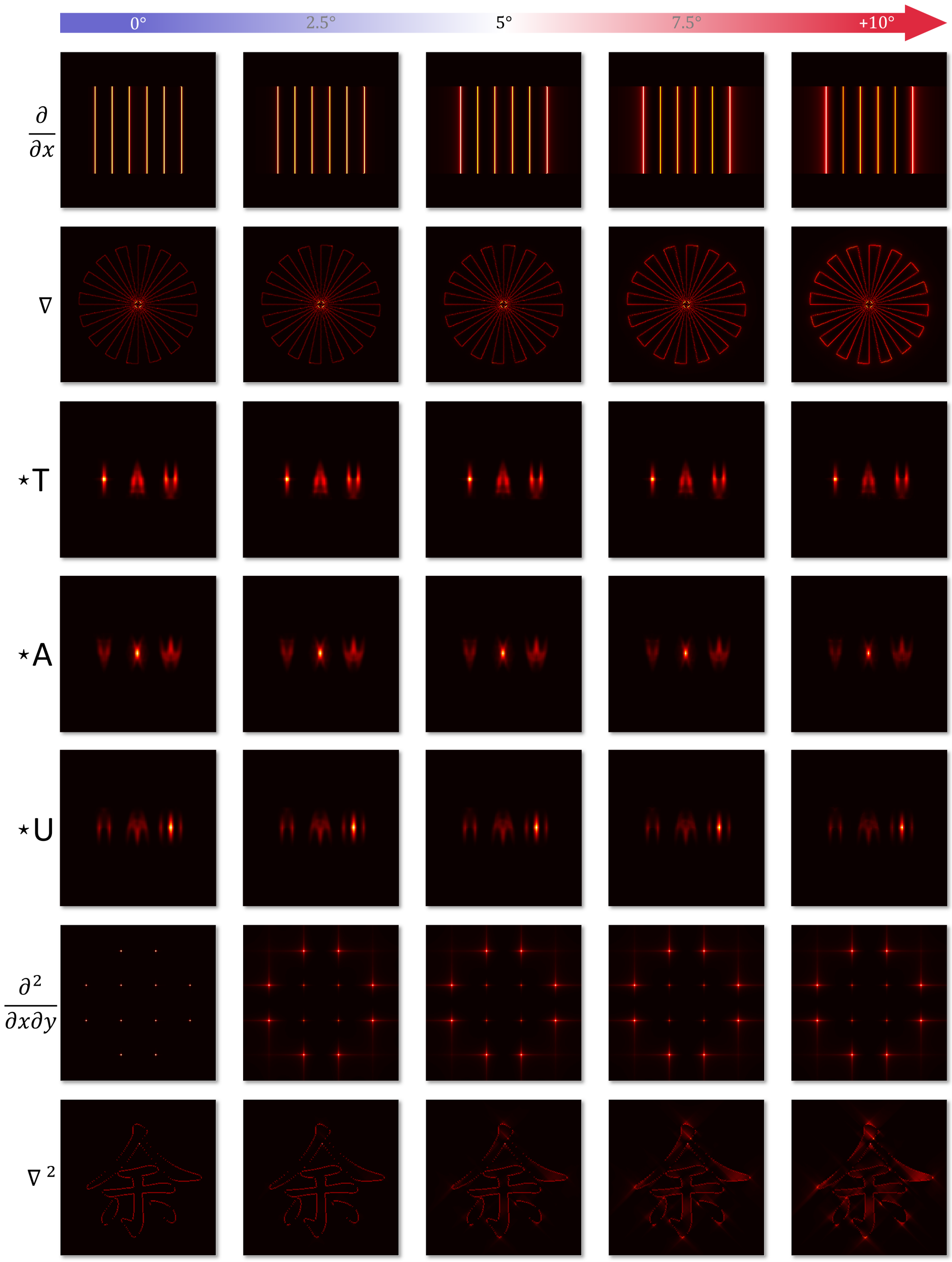

Fig. S19 Robustness of meta-operators to analyzer angle error. Simulated outputs as the analyzer angle error increases from 0° to 10°.

9. Broadband performance of meta-operators based on circular polarization multiplexing

Meta-operators that employ geometric phase with birefringent meta-atoms exhibit strong tolerance to wavelength variation. Although birefringence, and therefore polarization conversion efficiency, changes with wavelength, the geometric phase imparted to the cross-polarized component is wavelength independent. Because the output analyzer transmits only the cross-polarized light, the residual co-polarized component is rejected. From a wave-optics perspective, this filtering suppresses coherent interference between co-polarized components and supports clean image formation under off-design illumination.

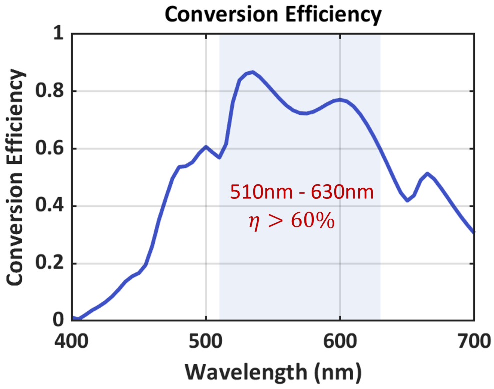

Fig. S20 Spectral dependence of cross-polarized conversion efficiency. Simulated efficiency from 400 to 700 nm for the birefringent meta-atom used in this work (length = 235 nm, width = 115 nm).

To characterize wavelength dependence, we simulated the cross polarized conversion efficiency of the birefringent meta-atom used in this paper (length = 235 nm, width = 115 nm). As shown in Fig. S20, the efficiency exceeds 60% from 510 to 630 nm, indicating effective operation across a large range of the visible spectrum.

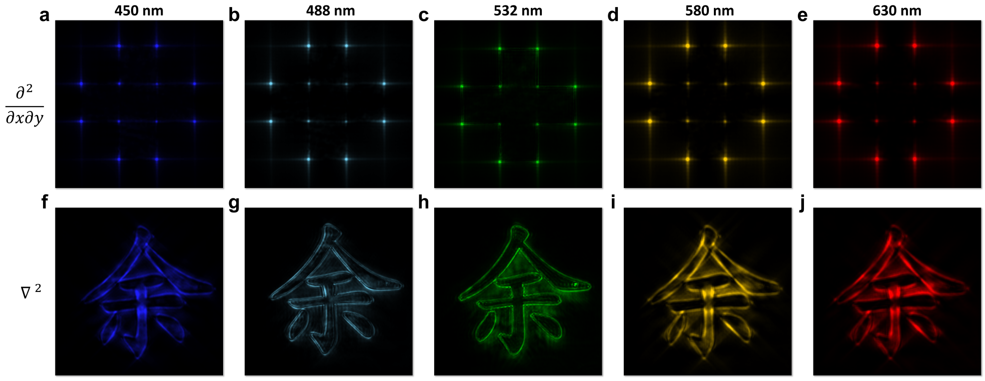

Fig. S21 Experimental demonstration of broadband robustness in meta-operators based on circular polarization multiplexing. (a–e) Vertex detection meta-operator applied to a cross pattern. (f–j) Laplacian meta-operator applied to the Chinese character *YU*. Both meta-operators were designed for 532 nm and tested at 450, 488, 532, 580 and 630 nm. (Laser source: SuperK FIANIUM, NKT Photonics).

We further validated broadband behavior experimentally. The same vertex detection and Laplacian meta-operators, designed for 532 nm, were tested at 450, 488, 532, 580 and 630 nm. As shown in Fig. S21, the outputs preserve the intended processing function across all five wavelengths. These measurements confirm the practical broadband robustness of meta-operators based on circular polarization multiplexing in the visible range.

10. Experiment setups

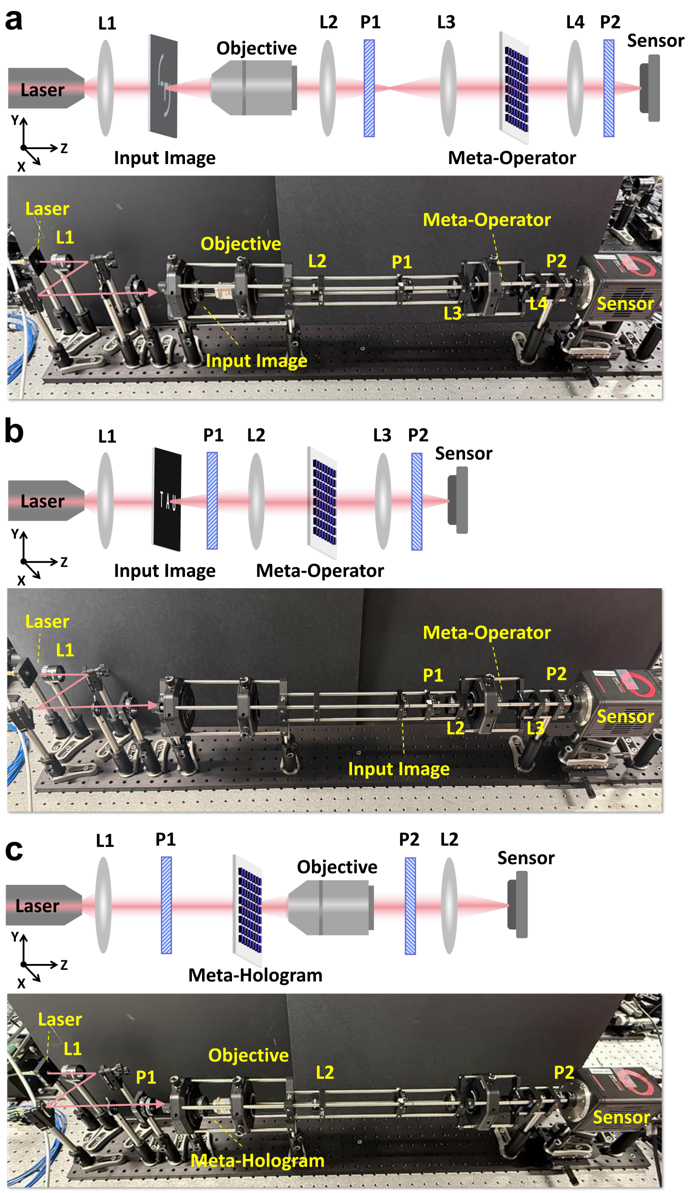

Fig. S22 Experimental setups. (a) Optical setup for evaluating meta-operators performing 1D and 2D first-order differentiation, vertex detection, and Laplacian differentiation. (b) Optical setup for evaluating meta-operators performing cross-correlation. (c) Optical setup for evaluating complex meta-holograms.

The experimental validation of the meta-operators and complex meta-holograms was conducted using the optical setups illustrated in Fig. S22.

The setup in Fig. S22a was used to evaluate the performance of 1D and 2D first-order differentiation, vertex detection, and Laplacian differentiation meta-operators. A collimated 532 nm laser beam, generated using a Raman laser (WiTec, Oxford Instruments), was expanded and collimated using an achromatic lens (L1, AC254-050-A-ML, Thorlabs). A pinhole was placed to control the beam size. The collimated beam then illuminated the test samples containing the input image patterns, and the transmitted light was collected by an objective lens (EC EPIPLAN-NEOFLUAR 2.5×/0.06, ZEISS) and an achromatic doublet (L2, AC254-150-A, Thorlabs), which acted as a tube lens to form an intermediate image. A polarizer (P1, LPVISE100-A, Thorlabs), positioned near the intermediate image plane, ensured equal amplitude in different polarization components before the beam entered the $4f$ system (L3, L4: AC254-075-AB-ML, Thorlabs), where the meta-operators were placed at the Fourier plane. The processed images were then projected onto a camera sensor (ORCA-Fusion C14440-20UP, Hamamatsu). A second polarizer (P2, LPVISE100-A, Thorlabs), positioned between the $4f$ system and the camera, enabled interference between the x- and y-polarized components for accurate image reconstruction.

The setup in Fig. S22b was used to evaluate the cross-correlation meta-operators. It followed a similar configuration to Fig. S22a but omitted the objective and tube lens to maintain unit magnification. The $4f$ system (L2, L3: AC254-075-AB-ML, Thorlabs) was directly employed to capture the input images, with the test samples positioned at the object plane of the $4f$ system for the cross-correlation operation.

The setup in Fig. S22c was used to characterize the complex meta-holograms. The same 532 nm laser was expanded and collimated using an achromatic lens (L1). A polarizer (P1) ensured equal intensity of the x- and y-polarized components before illuminating the meta-hologram. The modulated light fields at different depths were imaged using an objective lens (EC EPIPLAN-NEOFLUAR 10×/0.25, ZEISS) and an achromatic doublet (L2, AC254-150-A, Thorlabs), forming an intermediate image. This image was then relayed through a $4f$ system to the camera sensor. A second polarizer (P2) was placed before the camera to facilitate interference between the modulated polarization components, enabling depth-resolved holographic reconstruction.

11. TiO_2 metasurfaces fabrication

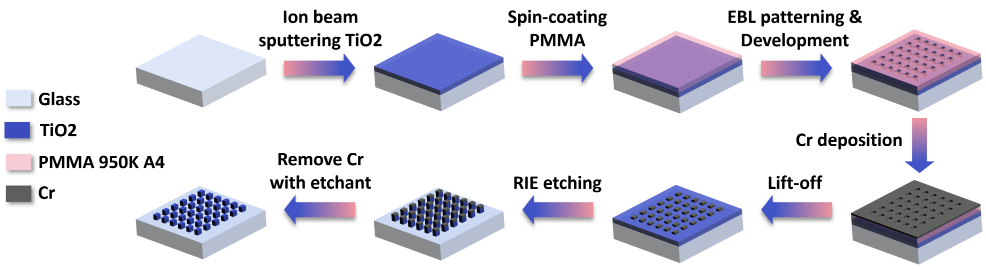

Fig. S23 Fabrication process flow of the TiO_2 metasurface.

The process flow of our metasurface fabrication is shown in Fig. S23 [4]. To fabricate the nanostructures on metasurface, a 600 nm thick TiO_2 layer was first deposited onto a 400 µm thick float glass substrate coated with 15 nm thick indium tin oxide (Präzisions Glas & Optik GmbH, CEC500S) via ion beam sputtering (Cutting Edge Coatings Navigator 700). A 250 nm thick layer of polymethyl methacrylate (PMMA) 950K A4 positive electron-beam resist was spin-coated onto the substrate, followed by baking at 180°C for 90 seconds. To facilitate alignment and prevent charge accumulation during electron-beam (e-beam) lithography, a 20 nm aluminum (Al) layer was deposited via e-beam evaporation (Instrumentti Mattila IM-9912). The pre-designed metasurface are convert to 2D mask pattern using Gdspy module in Python. The patterns were then exposed on the resist using an e-beam lithography system (Vistec EPBG5000pES). After exposure, the Al film was removed by immersion in an AZ351B : deionized water (1:5) solution. The samples were subsequently developed in a MIBK : IPA (1:3) solution. A 50 nm chromium (Cr) layer was deposited by e-beam evaporation to serve as a hard mask. After a 15-hour lift-off process in remover 1165, the patterns were transferred onto the Cr mask. These patterns were then etched into the TiO_2 film using reactive ion etching (Oxford Plasmalab System 100) with a gas mixture of SF_6 (5 sccm) and Ar (15 sccm), under a power of 125W at 30 mTorr and a temperature of 21°C. Finally, the Cr layer was removed using a Cr etchant solution (Sigma-Aldrich).

12. Test samples fabrication

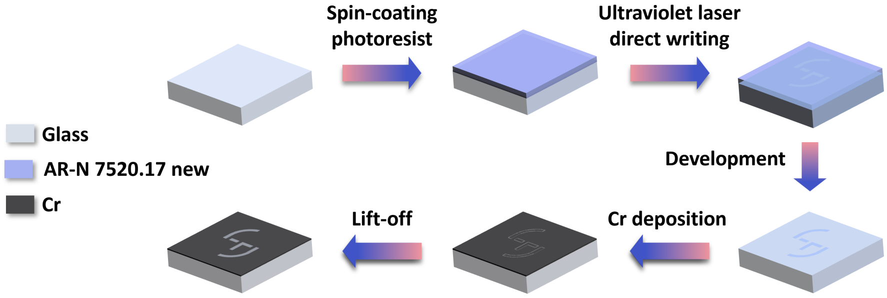

Fig. S24 Fabrication process flow of test samples with patterned images used for all-optical image processing experiments.

Fig. S24 illustrates the fabrication process flow for the test samples used in the meta-operator-based image processing experiments. A glass substrate was sequentially coated with two layers of photoresist (AR-N 7520.17 new) by spin-coating at 4000 rpm, with each layer baked at 85°C for 1 minute. The image patterns, shown in Fig. S25, were directly written onto the photoresist using a laser direct writing system (Heidelberg Instrument µPG 501). After development in AR 300-47, a 60 nm chromium (Cr) layer was deposited via electron-beam evaporation (Instrumentti Mattila IM-9912). The patterns were then transferred into a transparent aperture through a lift-off process using AR 300-73 at 50°C, enabling amplitude modulation of the incident light. The fabricated test samples are presented in Fig. S26.

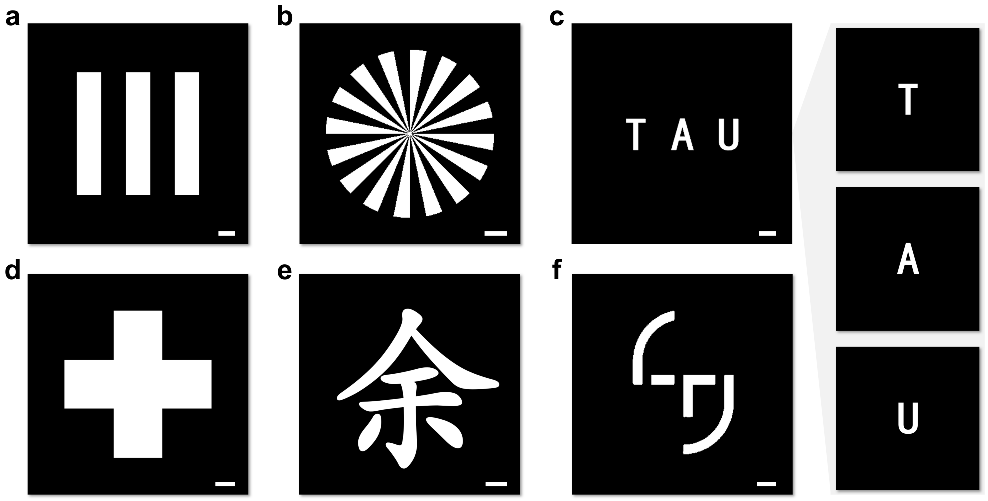

Fig. S25 Target patterns used in all-optical image processing experiments. (a) Bar pattern. (b) Spoke pattern. (c) *TAU* word pattern. (d) Cross pattern. (e) Chinese character *YU*. (f) Tampere University logo pattern. Scale bars: 200 µm.

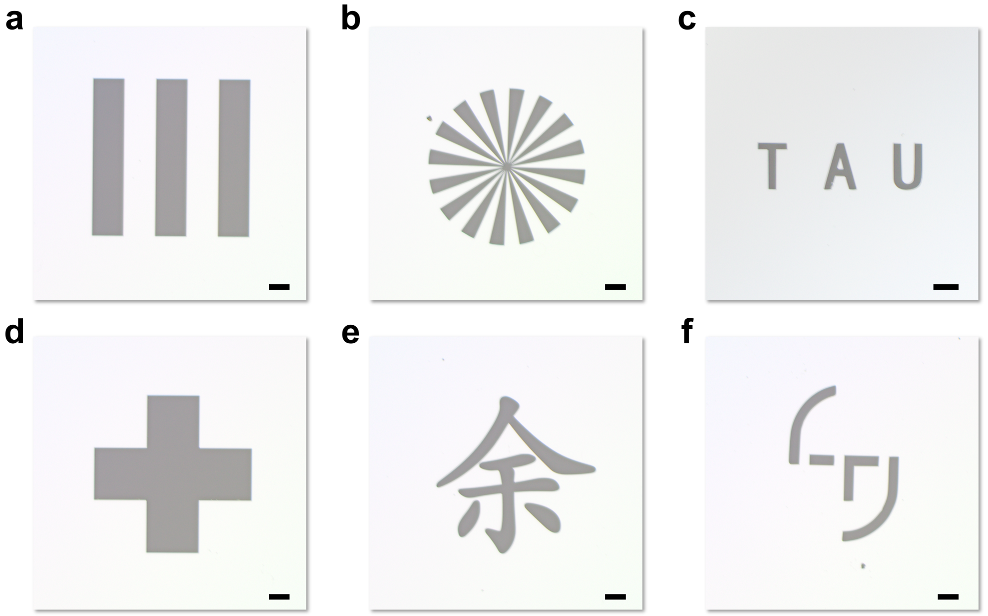

Fig. S26 Optical microscope images of test samples with patterns shown in Fig. S25. Scale bars: 200 µm.

13. Metalens-based $4f$ configuration for planar optical processor

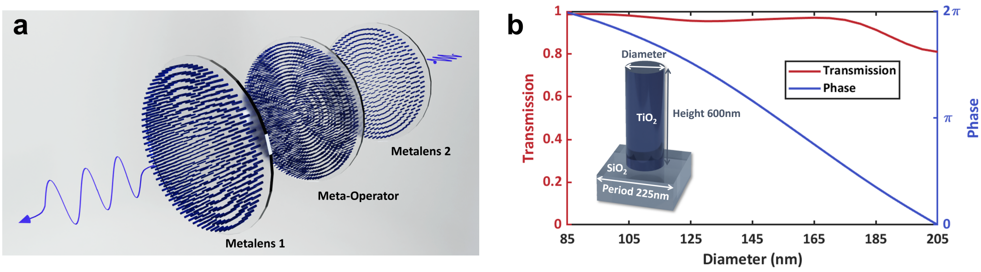

Fig. S27 Conceptual metalens-based $4f$ configuration. (a) Planar optical processor composed of two metalenses and a central meta-operator at the Fourier plane. (b) Example isotropic meta-atom design enabling polarization-insensitive phase control with high transmission.

To explore future integration possibilities, we present a conceptual, fully planar $4f$ relay that replaces the bulk lenses with high-efficiency metalenses and places the meta-operator at their common Fourier plane (Fig. S27a). Metalenses constructed from isotropic meta-atoms (example in Fig. S27b) provide polarization-insensitive, high-transmission phase control and can realize the Fourier transform required by the relay. In this configuration, frequency-domain processing is preserved while the system thickness is reduced.

Although this architecture offers a promising route toward miniaturization, it entails several practical tradeoffs. Short focal lengths, which are desirable for compact integration, limit the aperture and can cause vignetting, restricting the input field. Increasing the aperture to mitigate vignetting raises the numerical aperture (NA) and may introduce deviations from the ideal Fourier transform, particularly off axis. Nevertheless, advances in metalenses with large aperture and high numerical aperture, optimized for Fourier optics, continue to improve feasibility [5].

We also consider the polarization elements required for integration. Although an input polarizer and an output analyzer are required to enable interference between the two orthogonal polarization channels, the tolerance requirements are not stringent. As shown by our analysis of polarization misalignment (Section 8), the proposed operators are robust to angular misalignment and incomplete polarization conversion. Consequently, high precision components are unnecessary. Thin film polymer linear polarizers are sufficient and can be integrated with the metalenses or the metasurface layer. In addition, metasurface polarizers with high efficiency for prescribed polarization states have been demonstrated [6], which offers a promising route toward fully planar integration.

These considerations point to feasible paths toward compact, planar implementations of the $4f$ processor using metasurfaces.

14. Comparison with representative metasurface-based approaches for analog image processing

To clarify the advantages of the proposed platform, we compare it with representative metasurface-based methods recently explored for analog image processing. While many approaches have been proposed, the predominant recent directions can be grouped into four categories, comprising metasurface-based shearing interference, incidence-dependent resonant metasurfaces, diffractive optical processors, and complex transfer function metasurfaces, to which our method belongs, as summarized in Fig. S28.

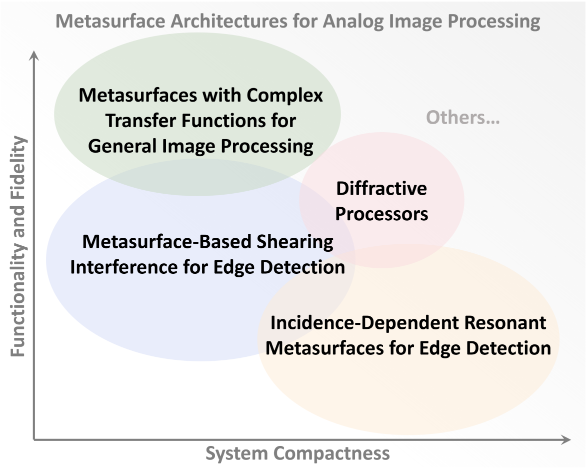

Fig. S28 Representative metasurface strategies for analog image processing.

Metasurface-based shearing interference exploits the photonic spin Hall effect in birefringent structures to create spatially shifted replicas of the input wavefront [7–10]. Interference between the displaced copies enhances edges along prescribed directions (lateral [8], radial [9], or azimuthal [10]) set by the metasurface design. Implementations are typically placed at a Fourier plane and can yield high-contrast edge features. Because the operation relies on geometric phase, the response is inherently broadband. However, the functionality is limited to directional differentiation and cannot be extended to arbitrary operators or complex transfer functions.

Another prominent approach to edge detection employs metasurfaces whose transmission depends on the angle of incidence [11–15]. By exploiting angle-selective resonances of the meta-atoms, these devices act as spatial frequency filters that transmit high spatial frequencies while suppressing lower ones. A key advantage is system compactness, since the element can be placed directly in the optical path rather than at a Fourier plane. However, resonance dispersion typically limits usable spectral bandwidth, and fine control over the spatial frequency passband is difficult. High spatial frequency components may also be partially reflected or distorted, and implementing arbitrary complex transfer functions remains challenging.

Diffractive processors, often termed diffractive neural networks, consist of multiple cascaded diffractive layers that encode learned phase profiles optimized for a target task [16–18]. These systems are compact, passive, and scalable. However, they are data driven and primarily suited to classification or other task specific imaging. The learned masks do not directly implement analytically specified operations, and changing the operation generally requires sophisticated retraining and redesign. Consequently, these systems provide limited analytical control over the implemented function, with reduced transparency and reproducibility compared with approaches based on explicit, mathematically defined operators.

Metasurfaces engineered to realize complex transfer functions provide a general and rigorous framework for analog optical computing. When placed at the Fourier plane, they implement analytically specified operators through spatial frequency modulation, enabling a broad range of image processing tasks. Although Fourier plane operation is required, these systems offer high fidelity, interpretability, and broad applicability. The principal challenge is realizing accurate complex modulation at high resolution for fine control of spatial frequency components, for which a few design strategies have been explored. One established strategy is structural tuning, in which both amplitude and phase are controlled by tailoring complex meta-atom geometries [19–22]. The reliance on intricate geometry introduces a high-dimensional design space with many structural degrees of freedom, often requiring extensive parameter sweeps or inverse design to identify viable cells. Such unit cells typically have a larger footprint, which reduces sampling density and can lower modulation efficiency. Moreover, the response is largely resonance driven and therefore strongly dispersive, making broadband operation difficult to realize. A second approach employs geometric phase, where rotation of birefringent meta-atoms sets the phase profile and the amplitude is determined by polarization conversion efficiency governed by the meta-atom geometry [23–25]. This method uses simple unit cell geometries, enables high spatial resolution, and is comparatively easy to fabricate. However, because amplitude control is tied to geometry dependent conversion that is strongly dispersive, performance degrades away from the design wavelength, which limits broadband applicability.

The proposed method in this work uses double-phase encoding that leverages controlled interference between two orthogonal polarization channels. Rather than relying on structural complexity or amplitude control based on polarization conversion efficiency, the metasurface encodes independent phase delays for the two polarizations. An output analyzer then combines the channels to synthesize an optical field with jointly modulated amplitude and phase, thereby enabling arbitrary complex transfer functions with simple, high-efficiency birefringent meta-atoms. Compared with structural tuning, this avoids large, intricate unit cells, supports higher spatial resolution, and improves modulation efficiency. It also reduces design complexity, since only independent phase control for two polarizations is required, rather than jointly engineering amplitude and phase. Relative to geometric phase designs that achieve amplitude control through polarization conversion efficiency, the approach offers similar structural simplicity and high resolution. Moreover, in the circular polarization multiplexing configuration, geometric phase fixes the phase profile irrespective of wavelength. As a

result, the synthesized complex transfer function remains stable across a broad spectral range.

In summary, these comparisons indicate that the proposed platform combines simple, high efficiency unit cells with mathematically specified Fourier domain operators. In configurations based on circular polarization multiplexing, it also demonstrates broadband operation. These features provide a flexible and scalable route to high-fidelity, general-purpose analog optical processing.

References

[1] Goodman, J.W.: Introduction to Fourier Optics. W.H. Freeman, New York (2017)

[2] Matsushima, K., Shimobaba, T.: Band-limited angular spectrum method for numerical simulation of free-space propagation in far and near fields. Optics Express **17**(22), 19662–19673 (2009)

[3] Hore, A., Ziou, D.: Image quality metrics: Psnr vs. ssim. In: 2010 20th International Conference on Pattern Recognition, pp. 2366–2369 (2010). IEEE

[4] Yang, W., Zhou, J., Tsai, D.P., Xiao, S.: Advanced manufacturing of dielectric meta-devices. Photonics Insights **3**(2), 04–04 (2024)

[5] Liu, W., Li, Z., Cheng, H., Tang, C., Li, J., Zhang, S., Chen, S., Tian, J.: Metasurface enabled wide-angle fourier lens. Advanced Materials **30**(23), 1706368 (2018)

[6] Wang, S., Wen, S., Deng, Z.-L., Li, X., Yang, Y.: Metasurface-based solid poincaré sphere polarizer. Physical Review Letters **130**(12), 123801 (2023)

[7] Liu, S., Chen, S., Wen, S., Luo, H.: Photonic spin hall effect: fundamentals and emergent applications. Opto-Electronic Science **1**(7), 220007–1 (2022)

[8] Zhou, J., Qian, H., Chen, C.-F., Zhao, J., Li, G., Wu, Q., Luo, H., Wen, S., Liu, Z.: Optical edge detection based on high-efficiency dielectric metasurface. Proceedings of the National Academy of Sciences **116**(23), 11137–11140 (2019)

[9] Zhou, J., Qian, H., Zhao, J., Tang, M., Wu, Q., Lei, M., Luo, H., Wen, S., Chen, S., Liu, Z.: Two-dimensional optical spatial differentiation and high-contrast imaging. National Science Review **8**(6), 176 (2021)

[10] Yu, L., Shevtsov, S., Singh, H.J., Kazansky, P.G., Caglayan, H.: Multifunctional meta-optic azimuthal shear interferometer. Nano Letters **25**(18), 7419–7425 (2025)

[11] Guo, C., Xiao, M., Minkov, M., Shi, Y., Fan, S.: Photonic crystal slab laplace operator for image differentiation. Optica **5**(3), 251–256 (2018)

[12] Kwon, H., Sounas, D., Cordaro, A., Polman, A., Alù, A.: Nonlocal metasurfaces for optical signal processing. Physical Review Letters **121**(17), 173004 (2018)

[13] Zhou, Y., Zheng, H., Kravchenko, I.I., Valentine, J.: Flat optics for image differentiation. Nature Photonics **14**(5), 316–323 (2020)

[14] Cotrufo, M., Sulejman, S.B., Wesemann, L., Rahman, M.A., Bhaskaran, M., Roberts, A., Alù, A.: Reconfigurable image processing metasurfaces with phase-change materials. Nature Communications **15**(1), 4483 (2024)

[15] Pearson, P., Roberts, G., Faraon, A.: Inverse-designed metasurfaces for multi-functional spatial frequency filtering. Optica **12**(7), 1090 (2025)

[16] Lin, X., Rivenson, Y., Yardimci, N.T., Veli, M., Luo, Y., Jarrahi, M., Ozcan, A.: All-optical machine learning using diffractive deep neural networks. Science **361**(6406), 1004–1008 (2018)

[17] Işıl, Ç., Gan, T., Ardic, F.O., Mentesoglu, K., Digani, J., Karaca, H., Chen, H., Li, J., Mengu, D., Jarrahi, M., *et al.*: All-optical image denoising using a diffractive visual processor. Light: Science & Applications **13**(1), 43 (2024)

[18] Hu, J., Mengu, D., Tzarouchis, D.C., Edwards, B., Engheta, N., Ozcan, A.: Diffractive optical computing in free space. Nature Communications **15**(1), 1525 (2024)

[19] Wang, Q., Zhang, X., Xu, Y., Gu, J., Li, Y., Tian, Z., Singh, R., Zhang, S., Han, J., Zhang, W.: Broadband metasurface holograms: toward complete phase and amplitude engineering. Scientific Reports **6**(1), 32867 (2016)

[20] Jia, S.L., Wan, X., Su, P., Zhao, Y.J., Cui, T.J.: Broadband metasurface for independent control of reflected amplitude and phase. AIP Advances **6**(4) (2016)

[21] Jiang, Q., Cao, L., Huang, L., He, Z., Jin, G.: A complex-amplitude hologram using an ultra-thin dielectric metasurface. Nanoscale **12**(47), 24162–24168 (2020)

[22] Deng, L., Li, Z., Guan, Z., Tao, J., Li, G., Zhu, X., Dai, Q., Fu, R., Zhou, Z., Yang, Y., *et al.*: Full complex-amplitude engineering by orientation-assisted bilayer metasurfaces. Advanced Optical Materials **11**(9), 2203095 (2023)

[23] Overvig, A.C., Shrestha, S., Malek, S.C., Lu, M., Stein, A., Zheng, C., Yu, N.: Dielectric metasurfaces for complete and independent control of the optical amplitude and phase. Light: Science & Applications **8**(1), 92 (2019)

[24] Ren, H., Fang, X., Jang, J., Bürger, J., Rho, J., Maier, S.A.: Complex-amplitude metasurface-based orbital angular momentum holography in momentum space. Nature Nanotechnology **15**(11), 948–955 (2020)

[25] Fu, W., Zhao, D., Li, Z., Liu, S., Tian, C., Huang, K.: Ultracompact meta-imagers for arbitrary all-optical convolution. Light: Science & Applications **11**(1), 62 (2022)
